# Magnetic Resonance Angiography Shows Increased Arterial Blood Supply Associated with Murine Mammary Cancer

**DOI:** 10.1155/2019/5987425

**Published:** 2019-01-17

**Authors:** Devkumar Mustafi, Abby Leinroth, Xiaobing Fan, Erica Markiewicz, Marta Zamora, Jeffrey Mueller, Suzanne D. Conzen, Gregory S. Karczmar

**Affiliations:** ^1^Department of Radiology, The University of Chicago, Chicago, Illinois 60637, USA; ^2^Department of Pathology, The University of Chicago, Chicago, Illinois 60637, USA; ^3^Department of Medicine, Section of Hematology and Oncology, The University of Chicago, Chicago, Illinois 60637, USA

## Abstract

Breast cancer is a major cause of morbidity and mortality in Western women. Tumor neoangiogenesis, the formation of new blood vessels from pre-existing ones, may be used as a prognostic marker for cancer progression. Clinical practice uses dynamic contrast enhanced magnetic resonance imaging (DCE-MRI) to detect cancers based on increased blood flow and capillary permeability. However, DCE-MRI requires repeated injections of contrast media. Therefore we explored the use of noninvasive time-of-flight (TOF) MR angiography for serial studies of mouse mammary glands to measure the number and size of arteries feeding mammary glands with and without cancer. Virgin female C3(1) SV40 TAg mice (n=9), aged 18-20 weeks, were imaged on a 9.4 Tesla small animal scanner. Multislice T_2_-weighted (T2W) images and TOF-MRI angiograms were acquired over inguinal mouse mammary glands. The data were analyzed to determine tumor burden in each mammary gland and the volume of arteries feeding each mammary gland. After* in vivo* MRI, inguinal mammary glands were excised and fixed in formalin for histology. TOF angiography detected arteries with a diameter as small as 0.1 mm feeding the mammary glands. A significant correlation (r=0.79; p< 0.0001) was found between tumor volume and the arterial blood volume measured in mammary glands. Mammary arterial blood volumes ranging from 0.08 mm^3^ to 3.81 mm^3^ were measured. Tumors and blood vessels found on* in vivo* T2W and TOF images, respectively, were confirmed with* ex vivo* histological images. These results demonstrate increased recruitment of arteries to mammary glands with cancer, likely associated with neoangiogenesis. Neoangiogenesis may be detected by TOF angiography without injection of contrast agents. This would be very useful in mouse models where repeat placement of I.V. lines is challenging. In addition, analogous methods could be tested in humans to evaluate the vasculature of suspicious lesions without using contrast agents.

## 1. Introduction

Breast cancer in humans is associated with increased blood supply and capillary permeability [1]. Typically, mammary vasculature is detected and evaluated using dynamic contrast enhanced (DCE) MRI [2]. This method requires rapid imaging following an I.V. injection of contrast media. Increased blood flow in cancers results in a greater rate of uptake of contrast media and more rapid enhancement in MR images. Changes in signal intensity following contrast media injection can be analyzed to determine contrast media uptake and to calculate physiological parameters relating to blood flow and permeability [2, 3].

While DCE-MRI is the preferred method for clinical detection of human breast cancer [4, 5], the use of DCE-MRI for serial measurements in mouse models of breast cancer is challenging. DCE-MRI requires the repeated use of an I.V., which may lead to complications. The accumulation of contrast agents in certain tissues is also problematic as repeated doses of contrast media can cause adverse reactions, such as kidney damage, in subjects [6]. Furthermore, DCE-MRI requires rapid imaging with time resolution of 1–2 seconds; this means that signal-to-noise ratio and spatial resolution is limited [3].

Time-of-flight MR angiography is a noninvasive means of assessing mammary gland vasculature. Time-of-flight angiography (TOFA) detects blood flow perpendicular to the image slice based on decreases in the apparent T1 signal due to in-flow [7]. TOFA primarily detects relatively rapid flow through arteries. Larger arteries are detected based on their higher signal-to-noise ratio (SNR) [8]. TOFA is thought to be less informative than DCE-MRI because there is concern that TOFA cannot depict small vessels with low blood velocities [9, 10]. However, unlike DCE-MRI, TOFA does not use contrast agents and is not limited by temporal resolution. Thus TOFA can be acquired over a longer period of time, which allows high spatial resolution. Images in all planes with high spatial resolution can be acquired to detect blood flow in smaller arteries [11, 12].

Previous studies in this lab have utilized serial imaging studies of the SV40 TAg mouse model of human breast cancer to examine the initiation and progression of mammary cancers [13]. The sensitivity and specificity of detecting the disease can be further improved by focusing on the consequences of tumor angiogenesis: increased micro-vessel density with altered vascular characteristics [11, 12]. We propose that TOFA is a useful alternative to DCE-MRI for evaluating vasculature, but with less negative repercussions. Furthermore, these two types of MRI acquisition protocols should complement each other and could provide an improved understanding of the angiogenesis associated with cancer aggressiveness and growth.

The goal of the present research was to evaluate the quality of TOFA in mouse mammary glands, and to determine the range of sizes of arteries that could be detected. In addition, we tested whether TOFA detects changes in arterial blood supply associated with neoangiogenesis and growth of mammary cancer.

## 2. Materials and Methods

### 2.1. Animal

Nine FVB/N mice homozygous for the SV40 TAg transgene (originally provided as hemizygous TAg mice by Dr. Jeffrey E. Green of the National Cancer Institute's Mouse Models of Cancer Consortium) were weaned at 3 weeks of age as previously described [14]. These mice were sacrificed after* in vivo* MRI studies at 18-20 weeks of age. Mice were anesthetized prior to* in vivo* MR imaging, and anesthesia was maintained during imaging with 1-2% isoflurane. Temperature, heart rate, and respiration were monitored by SA instruments (Stony Brook, NY, USA), and the respiration rate was used to gate imaging. After* in vivo* imaging, mice were sacrificed by an overdose of isoflurane and cervical dislocation.

### 2.2. MRI Experiments

*In vivo *imaging: MR images were acquired on a 9.4 Tesla small animal scanner (Bruker, Ettlingen, Germany) with 11.6 cm inner diameter actively shielded gradient coils (maximum constant gradient strength for all axes: 230 mT/m). The mouse was placed supine on an animal holder and inserted into a 30 mm diameter quadrature volume coil (Rapid MR International, Columbus, OH, USA). Multislice RARE (Rapid Acquisition with Relaxation Enhancement) T_2_-weighted (T2W) images with fat suppression were acquired with the following parameters: TR/TE_effective_ = 4000/20 ms, field-of-view (FOV) = 25.6 mm × 19.2 mm, matrix size = 256 × 192, slice thickness = 0.5 mm, RARE factor = 4, and number of excitations (NEX) = 2. For the inguinal glands (left and right) two interleaved sets of images were acquired to cover the slice gaps of 1 mm and then combined together for a total of 62 slices.

For TOFA, a flow compensated gradient echo sequence with a short TR and thin slices was used to maximize inflow effects and depict flowing blood as a bright signal. Parameters for TOF with the same in-plane resolution as T2W images were TR/TE = 10/3 ms, flip angle = 60°, FOV = 25.6 mm × 19.2 mm, matrix size = 256 × 192, slice thickness = 0.5 mm, 61 slices, and NEX = 4. The acquisition time for each slice was 10.2 s and the total acquisition time for this TOF sequence was 10.4 minutes.

### 2.3. Histology

After* in vivo* MRI studies, the mammary glands were excised and placed in 10% formalin for tissue fixation for two weeks, then put in a histology cassette in ethanol for immediate histological processing and H&E staining of slices. An experienced breast pathologist (JM) evaluated the histological slides and tissue was classified as normal gland,* in situ*, or invasive cancers. H&E slides were then scanned using a fully automated Leica microscope (DM-6000B, Leica Microsystems, Weltzar, Germany) for visualization and H&E images were stored in TIFF format.

### 2.4. Data Analysis

The MRI data sets were processed and analyzed quantitatively using software written in IDL (Exelis VIS, Inc., Boulder, CO, USA). Amira 3D visualization and analysis software (FEI Visualization Sciences Group, Burlington, MA, USA) was used to create three-dimensional displays of the tumors and blood vessels. Based on criteria defined and published previously, we identified invasive tumors in the inguinal mammary glands from nine SV40 mice on T2W images [15]. Then regions-of-interest (ROIs) were manually traced around mammary cancers. Total tumor volume in each mammary gland was determined by combining the volumes of all cancer ROIs.

To determine the volumes of arteries feeding mammary glands, the left and right inguinal mammary gland were manually traced on T2W images first and the resulting ROIs were superimposed to the TOF images. Then, thresholds were set to select only for those pixels representing blood vessels. Using these pixels, the total volumes of arteries in the left and right mammary gland were calculated.

We calculated the volumes of the mammary cancers or blood vessels on each slice by multiplying the total cross sectional area by the slice thickness of 0.5 mm in both T2W and TOF images. All of the slice volumes were added together to estimate the final tumor volume and vessel volumes on each side of the mammary gland. The Pearson correlation test was performed to examine whether there is a linear relationship between mammary cancer volumes and blood volumes. The t-test was performed to determine whether there was a statistically significant difference for calculated parameters. A p-value of less than 0.05 was considered significant.

## 3. Results

Using TOFA, the smallest detectable artery on volume rendered images was 0.005 mm^3^. In this case, the slice thickness was the limiting factor in determining size. The average (± standard deviation) SNR of muscle in T2W images, measured over ROIs (n = 27), was 17.3±6.4. The average SNR of muscle in TOF images, measured over ROIs (n = 27), was 3.6±1.9. The average SNR of blood vessels in TOF, measured over ROIs (n = 27), was 61.1±22.0. The blood vessels had significantly higher SNR than muscle (p < 0.0004). This allowed for the detection of blood flow in smaller arteries.

**Figure 1:**
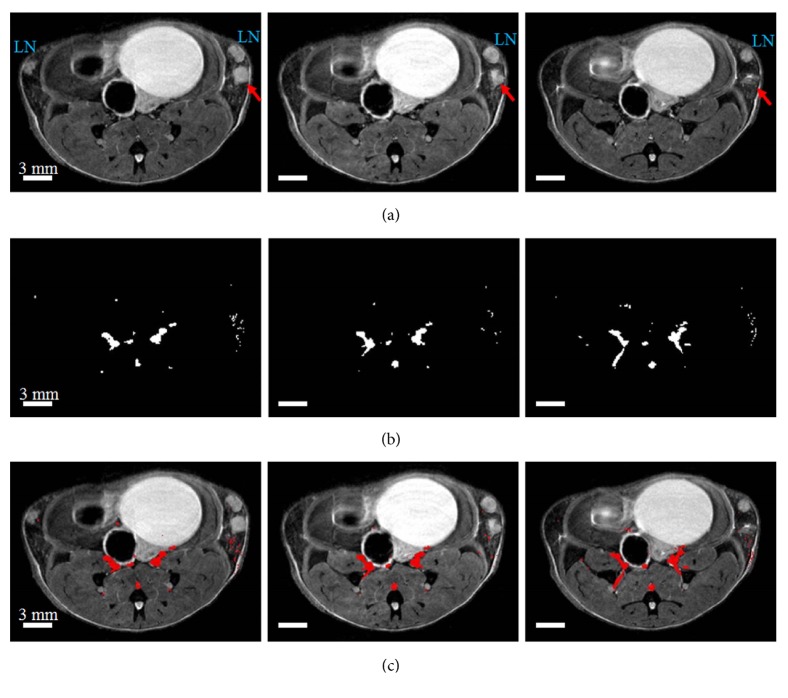
*In vivo* MR images of a SV40 TAg mouse of 19 weeks of age. The top panel (a) shows T2W images of the three central 0.5 mmm thick slices through inguinal mouse mammary glands. In all images lymph nodes (LN) are labeled and tumors are indicated by red arrows. The middle panel (b) shows the TOF images of the corresponding slices as seen in the top panel. The bottom panel (c) shows blood vessels, shown in red as in TOF images in the middle panel, and overlaid on T2W images. Scale bars in all images are shown.

Using TOFA and T2W images, we were able to measure arterial blood volumes.  Figure 1(a) shows three contiguous T2W images (left to right) of an SV40 TAg female mouse with a tumor (red arrow) developing beneath the lymph node (denoted as LN).  Figure 1(b) shows three TOF images from approximately the same slices as shown in Figure 1(a).  Figure 1(c) shows blood vessels, in red, overlaid on the T2W images. These images demonstrate that blood volume increases with cancer burden, as blood vessels are more abundant in the right gland, particularly beneath the tumor. In the left gland no cancer is evident and no blood vessels are detected.

**Figure 2:**
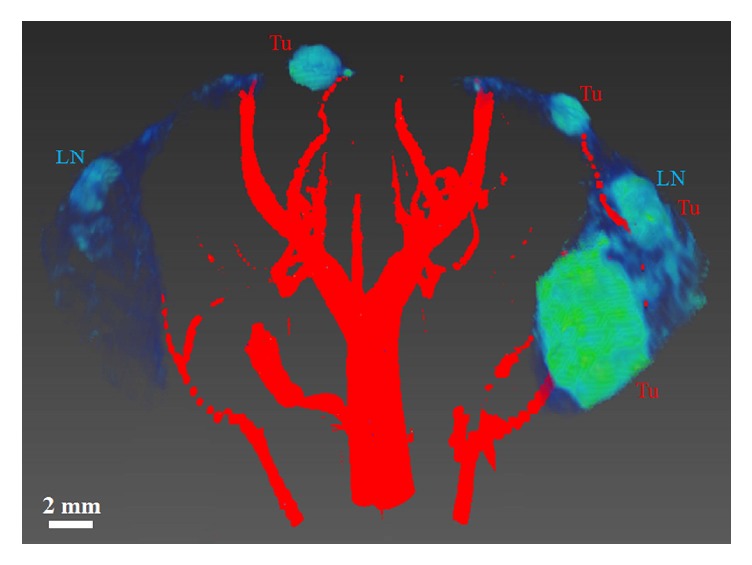
**Three dimensional volume rendered image shows mouse mammary glands and vessel densities of a SV40 TAg female mouse at 18 weeks of age.** Only the ROIs of both inguinal mammary glands are shown in blue-to-green color—images were acquired with a T2W RARE sequence. Blood vessels are shown in red—images of blood vessels were constructed from TOF datasets. Blood vessels in and near the right inguinal gland, compared to the left gland, grew significantly as invasive cancers developed. Lymph nodes (LN) and tumors (Tu) are labeled. A scale bar of 2 mm is also shown.

This qualitative relationship was visualized more easily with three-dimensional rendering, (using Amira 3D software).  Figure 2 shows a 2-dimensional depiction of the 3D volume-rendering of a SV40 TAg female mouse at 18 weeks of age. Greater arterial blood supply (in red) can be seen flowing towards the tumors (labeled “Tu”) in the right gland (blue-to-green). In comparison, the left inguinal gland shows neither cancer nor arteries. Visual inspection of volume-rendered images in 3D suggested differences in the organization of arteries associated with mammary cancer. Arteries leading to mammary glands with low or no tumor burden are well organized in a tree-like structure with alternating branches. Arteries leading to glands with higher tumor burdens do not follow this regular branching pattern. Instead the blood vessels grow in a clump directed towards the tumor and surrounding tissue.

**Figure 3:**
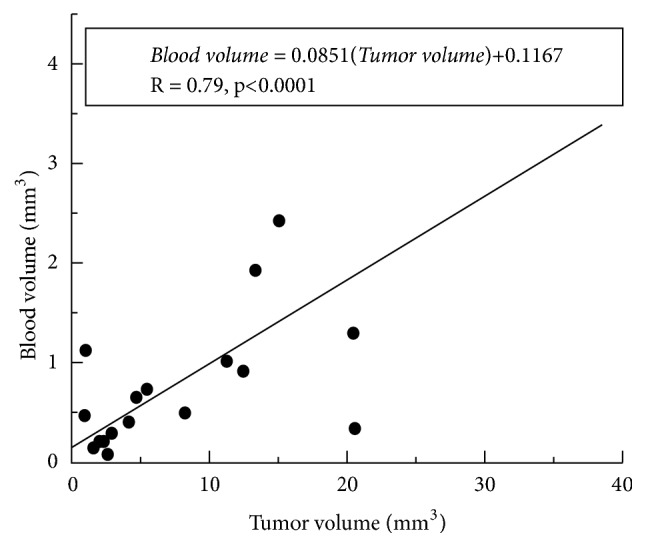
**Plot of blood volume as a function of tumor volume in SV40 TAg mice**. The scatter plot shows the relationship between the tumor and blood volumes for a total of nine SV40 TAg mice of 18-20 weeks of age. There was a strong positive correlation (r = 0.79, p < 0.0001) between the tumor volume and the blood volume as indicated in the plot.

A strong positive correlation (r = 0.79, p < 0.0001) was found between increasing tumor volume and blood volume, as seen in Figure 3. The best-fit straight line through the plot of tumor volume vs. blood volume is given by Blood-volume = 0.085*∗*Tumor-volume + 0.12.

**Figure 4:**
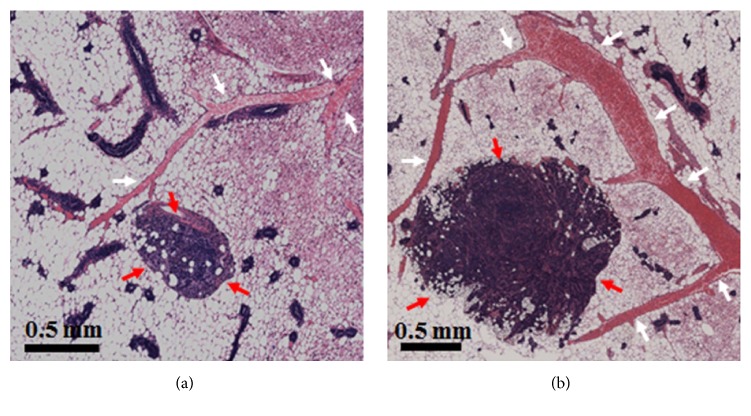
**Histological images of mouse mammary glands of a SV40 TAg mouse**. Hematoxylin and eosin- (H&E-) stained images of the excised (a) left and (b) right inguinal glands of a SV40 TAg mouse at 19 weeks of age are compared. Increased and more dilated blood vessels associated with the larger tumor are seen on the right inguinal gland (b) compared to the left gland (a). In both images tumors are indicated by red arrows, while blood vessels are indicated by white arrows. A scale bar of 0.5 mm in each image is also shown.

This result is consistent with histology.  Figure 4 depicts the histology of the mouse shown in Figure 2. This histology shows that the right gland (Figure 4(b)), which has a larger tumor and not only has more vessels but also more vascular complexity, as compared to the left gland (Figure 4(a)). An experienced breast pathologist (JM) identified cancers and blood vessels from H&E-stained slices. Previous studies in the lab correlated MRI-TOFA results with both H&E-stained and CD31-stained slices, demonstrating the usefulness of MRI-TOFA in the use of H&E stained and CD31 stained histology [16].

## 4. Discussion

This study found a strong positive correlation between arterial blood volume in the mammary gland and mammary cancer volume using TOFA. The correlation between vasculature and tumor aggressiveness has been previously studied and established, and antiangiogenic therapies have become an important option for anti-cancer therapies [7, 12, 17–20]. The results demonstrate the effectiveness of TOFA for qualitative and quantitative measurements of changes in vasculature as cancer develops.

High-resolution magnetic resonance angiography (MRA) offers a macroscopic view of the entire arterial supply of cancer, as documented in this study. MRA-based parameters may have diagnostic utility. In a previous blinded study of human brain cancer patients, a statistical analysis of the shapes of MRA-extracted blood vessels proved successful in separating benign from malignant disease in all cases on the basis of image analysis before lesion resection [21]. In contrast to perfusion imaging (e.g., DCE-MRI), high-resolution MRA shows the 3D anatomy of the arterial supply to cancers [22]. The major advantage of TOFA of mouse models lies in the ability to conduct repeated studies completely noninvasively, avoiding use of intravenous catheters and contrast agent injection. This makes TOFA a valuable tool for directly monitoring cancer development and the effectiveness of antiangiogenic therapies without injuring or sacrificing animals. The method used here does not readily provide quantitative measurements of blood flow, but more quantitative TOFA methods are available [23].

A major concern with TOF imaging has been that it cannot detect small arteries; this could result in poor sensitivity to neoangiogenesis associated with mammary cancer. However, in this study at 9.4 T, TOA reliably measured blood volumes as low as 0.08 mm^3^ and detected individual arteries as small as 5x10^−4^ mm^3^ in volume. The accuracy of arterial blood volume measurements in this study was limited primarily by the slice thickness of 0.5 mm, since in-plane resolution was 0.1 mm × 0.1 mm. In contrast to DCE-MRI, the TOF sequence does not require high temporal resolution. Therefore, more signal averaging can be used to acquire TOF images with higher spatial resolution, to detect even smaller arteries. Furthermore, TOF images can be acquired in both sagittal and coronal planes, in addition to the axial plane seen in the present study. This would allow evaluation of arterial blood flow in three-dimensions.

This study demonstrated a positive linear relationship (Figure 3) between the tumor growth and the local blood volume. The results suggest that blood volume in the mammary gland is approximately 8.5% of tumor volume. However, not all of the cancers identified in this study conformed to the approximately linear relationship between blood volume and tumor burden—there were several outliers. We are not able to determine yet whether this reflects biological variability or measurement error. Previous work suggests that neoangiogenesis is biologically variable in cancers [9].

Previous studies have correlated the number of vessels with aggressiveness in brain cancer [7, 17, 24]. To the best of our knowledge the present study is the first to demonstrate a strong correlation between tumor volume and local blood volume in mammary cancer, without use of contrast agents, noninvasively and* in-vivo* [2, 3]. The results demonstrate that TOFA can noninvasively monitor changes in vasculature during cancer development in mouse models, without the need to place I.V. lines and inject contrast agents. In addition, the present results provide a rationale for testing this approach in patients to reduce costs, avoid adverse effects of contrast agents, and improve image quality.

## Data Availability

The datasets used to support the findings of this study are available from the corresponding author upon request.
